# Convalescent plasma to treat COVID-19: clinical experience and efficacy

**DOI:** 10.18632/aging.202795

**Published:** 2021-03-18

**Authors:** Shiyao Pei, Xi Yuan, Zhimin Zhang, Run Yao, Yubin Xie, Minxue Shen, Bijuan Li, Xiang Chen, Mingzhu Yin

**Affiliations:** 1 Hunan Engineering Research Center of Obstetrics and Gynecological Disease, Xiangya Hospital, Central South University, Changsha 410008, Hunan Province, China; 2 Department of Dermatology, Hunan Engineering Research Center of Skin Heath and Disease, Xiangya Hospital, Central South University, Changsha 410008, Hunan Province, China; 3 Department of Blood Transfusion of Xiangya Hospital, Central South University, Changsha 410008, Hunan Province, China; 4 Department of Blood Transfusion Laboratory of Changsha Blood Center, Changsha 410008, Hunan Province, China

**Keywords:** COVID-19, convalescent plasma

## Abstract

The recent outbreak of COVID-19 in the world is currently a big threat to global health and economy. Convalescent plasma has been confirmed effective against the novel corona virus in preliminary studies. In this paper, we first described the therapeutic schedule, antibody detection method, indications, contraindications of the convalescent plasmas and reported the effectiveness of convalescent plasma therapy by a retrospective cohort study.

## INTRODUCTION

**Figure 1:**
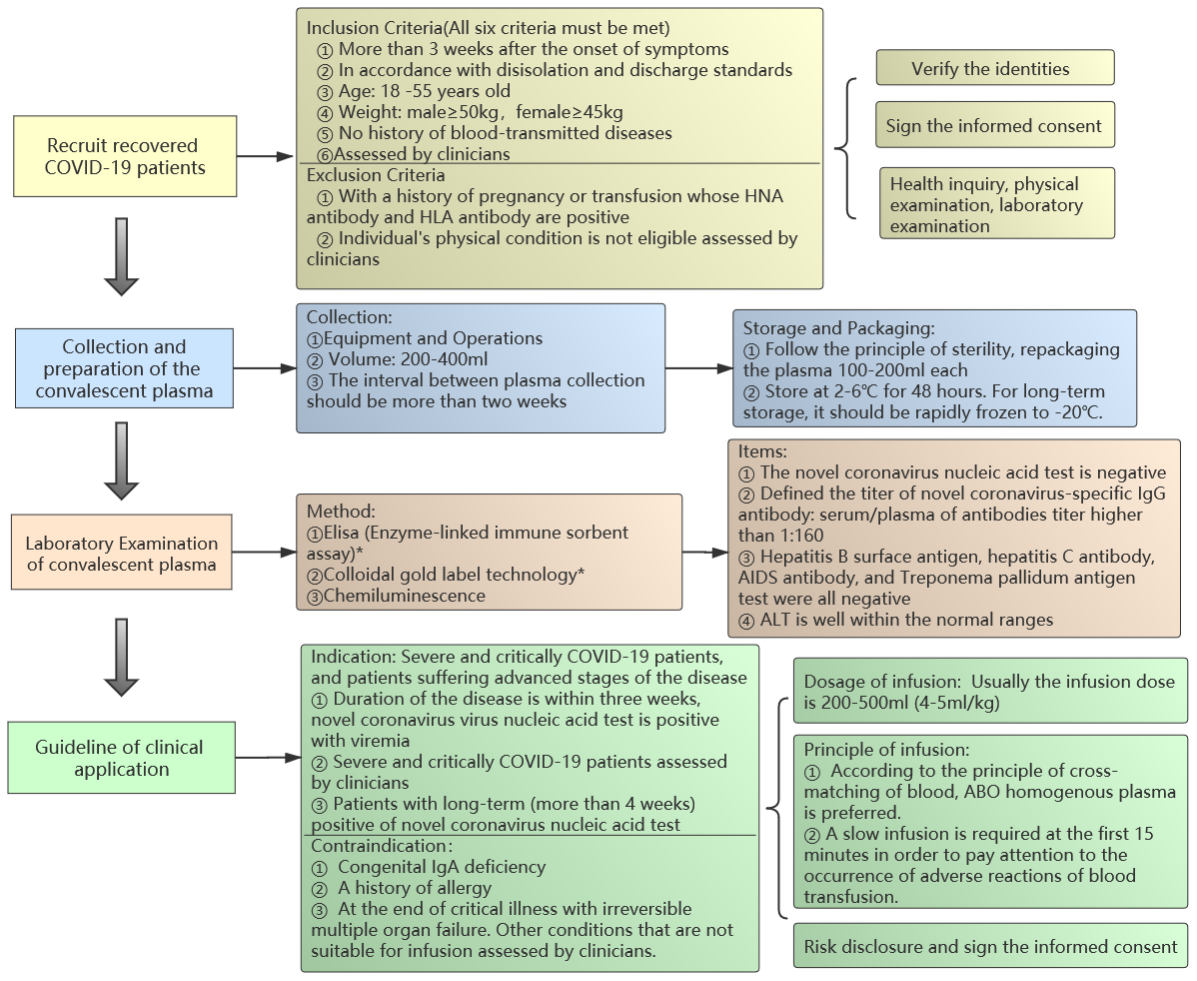
**The standardized flow chart of the convalescent plasma transfusion.**

In December 2019, pneumonia associated with a novel corona virus 2019 (COVID-19) caused an outbreak, which has posed significant threats to global health and economy [1]. As of 6th April 2020, the World Health Organization Situation Reported that this epidemic had spread to more than 180 countries with 1,113,758 confirmed cases, including 62,784 deaths [2]. It was reported that severely/critically ill case ratio was approximately 7-10% [3], while the current treatment strategy mainly rely on the supportive care since specific drugs of COVID-19 are still being researched. On March 4, 2020, in order to improve the therapeutic effect of COVID-19, the National Health Commission of the People’s Republic of China organized Chinese experts to make revisions of the “Clinical treatment of COVID-19 Convalescent Plasma (the second trial edition)” [4]. On March 24, 2020, FDA approved the testing of convalescent plasmas for patients with serious or immediately life-threatening COVID-19 infections [5]. To date, thousands of convalescent plasmas have been collected and remarkable efficacy has been achieved in severely and critically ill COVID-19 patients in China. In order to standardize the treatment of COVID-19 Convalescent Plasma and share the clinical experience with the world, we summarized the therapeutic schedule as follows (Figure 1).

## RESULTS

### Recruit recovered COVID-19 patients

1. Inclusion Criteria (All six criteria must be met)
  - More than 3 weeks after the onset of symptoms of the COVID-19 and complete resolution of symptoms at least 14 days prior to donation.
  - In accordance with relieved isolation and discharge standards following the latest version of the therapeutic schedule.
  - Age: 18 -55 years old.
  - Weight: male≥50kg, female≥45kg.
  - No history of blood-transmitted diseases.
  - Eligible donors must be assessed by clinicians according to treatment.
2. Exclusion Criteria
  - With a history of pregnancy or transfusion whose HNA antibody and HLA antibody are positive.
  - Individual’s physical condition is not eligible assessed by clinicians.
3. Verify the identities
4. Sign the informed consent
5. Health inquiry, physical examination, laboratory examination of blood samples (refer to technical operation procedures of blood station).

### Collection and preparation of the convalescent plasma

1. Collection
  - Equipment and Operations: fully automatic apheresis machine or a fully automatic blood cell separator (refer to technical operation procedures of blood station).
  - Volume: 200-400ml (The exact volume should be assessed by clinicians).
  - The interval between plasma collection should be more than two weeks.
2. Storage
  - Follow the principle of sterility, repackaging the plasma 100-200ml each.
  - Store at 2-6° C for 48 hours. For long-term storage, it should be rapidly frozen to -20° C.
3. Packaging: Labelling Requirements: refer to technical operation procedures of blood station.

### Laboratory examination of convalescent plasma

1. Method: Elisa (Enzyme-linked immune sorbent assay)*, Colloidal gold label technology*, Chemiluminescence.
  *Note: We recommend using ELISA to detect the novel coronavirus antibody titer since the colloidal gold method was not suitable for titer detection and the false negative rate was high. The analysis of 17 samples showed that the positive rate and sensitivity of ELISA were significantly better than colloidal gold (The specific data is shown in Supplementary Table 1).
2. Items
  - The novel coronavirus nucleic acid test is negative.
  - Defined the titer of novel coronavirus-specific IgG antibody: serum/plasma of antibodies titer higher than 1:160.
  - Hepatitis B surface antigen, hepatitis C antibody, HIV antibody, and Treponema pallidum antigen test were all negative.
  - Alanine aminotransferase is well within the normal ranges.

### Guideline of clinical application

**Figure 2:**
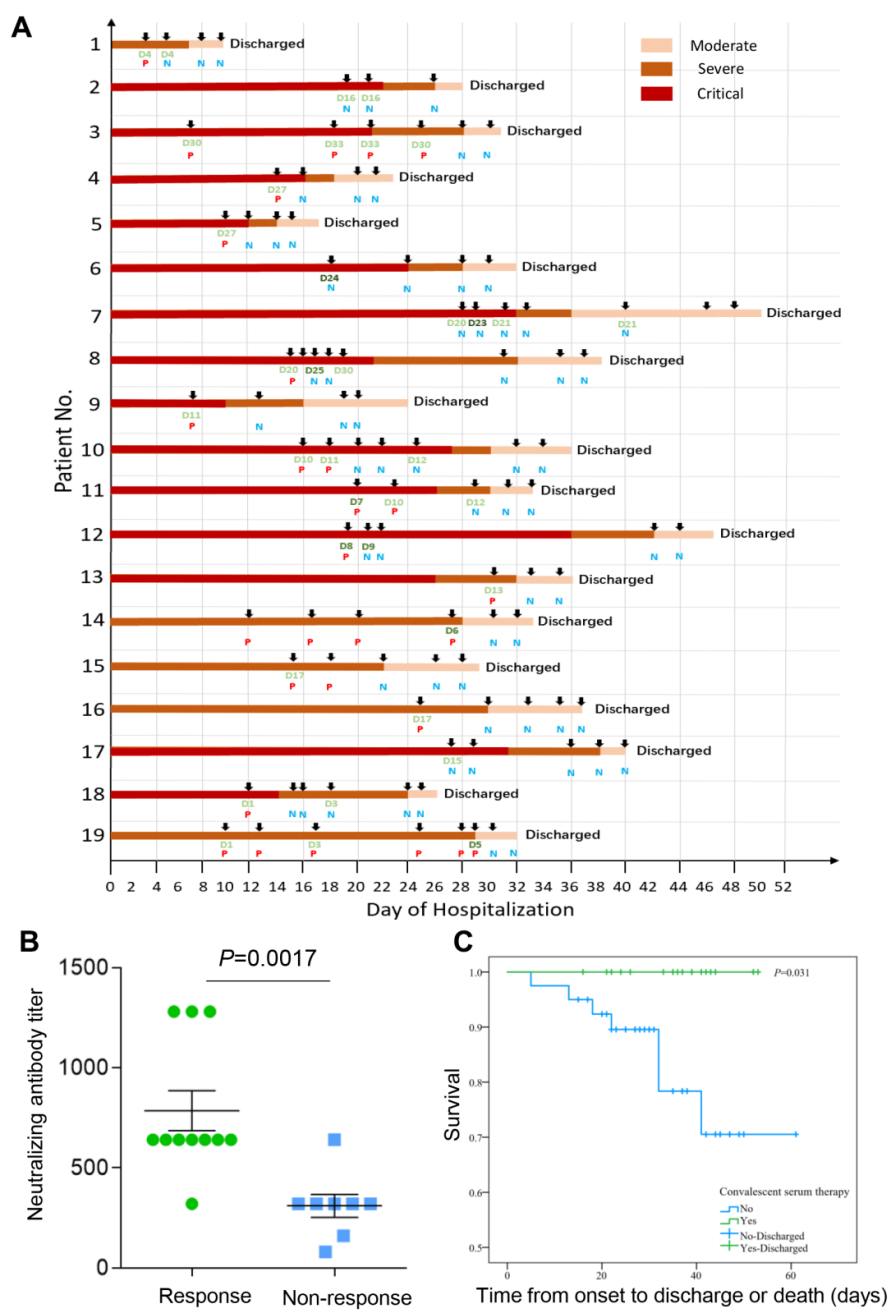
(**A**) Outcomes for individual patients included in 19 cases. Donor and receiver detail information see Supplementary Table 1; P, Nucleic acid test positive; N, Nucleic acid test negative; D, Donor patient (200ml); D, Donor patient (400ml). (**B**) The relationship between titer of neutralizing antibody from the donors and the therapy response of the COVID-19 patients. The plasma from the donors with a higher neutralizing antibody titer had a better treatment response (p=0.0017). The clinical symptoms were significantly improved and viral nucleic acid tests turned negative within five days after CP treatment was defined as “Response”, otherwise it was “Non-response”. (**C**) The survival curves of the exposure group and control group. All the 19 patients treated with CP transfusion in our study survived, and showed a significantly lower case-fatality rate compared to the control group (0% vs. 19%, *P*=0.031).

1. Indication: Severe and critically COVID-19 patients, and patients suffering advanced stages of the disease.
  - Duration of the disease is within three weeks, novel coronavirus virus nucleic acid test is positive with viremia.
  - Severely and critically ill COVID-19 patients assessed by clinicians.
  - Patients with long-term (more than 4 weeks) positive of novel coronavirus nucleic acid test (for details please refer to patient 2 in Figure 2).
2. Contraindication
  - Congenital IgA deficiency.
  - A history of allergy including plasma infusion, human plasma protein products, sodium citrate. Plasma inactivated by methylene blue virus is strictly prohibited in patients with methylene blue allergy. Other history of severe allergies and contraindications.
  - At the end of critical illness with irreversible multiple organ failure. Other conditions that are not suitable for infusion assessed by clinicians.
3. Dosage of infusion: According to the clinical status and the patient’s weight. Usually the infusion dose is 200-500ml (4-5ml/kg).
4. Principle of infusion
  - According to the principle of cross-matching of blood, ABO homogenous plasma is preferred.
  - A slow infusion is required at the first 15 minutes in order to pay attention to the occurrence of adverse reactions of blood transfusion.
5. Risk disclosure and sign the informed consent

### The retrospective cohort study of the convalescent plasma transfusion

The individual CP therapeutic process and outcomes of the 19 patients treated with CP transfusion were shown in Figure 2A. It can be clearly seen that symptoms in the 19 patients were all improved according to the different shades of color, which signifies the severity of the disease. Four critically ill patients with negative detection of viral nucleic acid (ID: 2, 6, 7, 17) also showed a good response to the CP treatment. By analyzing the titer of neutralizing antibody from the donors and the therapy response of the COVID-19 patients, we found that the plasma from the donors with a higher neutralizing antibody titer had a better treatment response (p=0.0017) (Figure 2B). Consistent with previous studies, CP treatment could improve the clinical outcomes through neutralizing viremia and decrease the viral load. According to the latest treatment guidelines, two consecutive negative viral nucleic acid tests can be regarded as the standard of discharge. Our results showed that the viral nucleic acid tests turned negative immediately after the CP treatment in the critically ill patients (ID: 4, 5, 8, 9, 10, 12, 18) and the moderately/severely ill patients with persistently positive detection of viral nucleic acid for more than three weeks (ID: 3, 13, 14, 15, 16, 19) (Figure 2A). All the 19 patients treated with CP transfusion in our study were survived, and showed a significantly lower case-fatality rate compared to the control group (0% vs. 19%, p=0.031). The survival curves of the exposure group and control group were shown in Figure 2C.

## DISCUSSION

The use of convalescent plasma has a long history. At the end of the 19th century, researchers found that recovery patients’ plasma was effective in diphtheria and tetanus patients. Use of convalescent plasma has been studied in outbreaks of other respiratory infections, including the 2003 SARS-CoV-1, 2009-2010 H1N1 influenza virus pandemic and the 2012 MERS-CoV epidemic [6]. Previous studies showed a shorter hospital stay and lower mortality with no adverse events or complications in patients who treated with convalescent plasma treatment than those who were not [7]. Additionally, viral load after convalescent plasma treatment was significantly lower on days 3, 5, and 7 after intensive care unit admission [8]. What’s more, we found that patients with long-term positive nucleic acid test of novel coronavirus turn negative earlier after convalescent plasma treatment than those who without convalescent plasma treatment. Furthermore, asymptomatic patients with hypoimmunity, such as the elderly, children and patients with underlying diseases such as diabetes, hepatitis, AIDS, heart disease, tuberculosis, malignant tumor, etc., preferred to use convalescent plasma once the nucleic acid test was positive.

In our study, 19 COVID-19 patients were treated with CP and recovered quickly from the disease. The clinical symptoms were significantly improved as the individual critical/severe illness condition turned to moderate after CP treatment. The viral shedding was minimized and the clinical conditions of these patients improved as indicated by the viral nucleic acid test. We also found that the CP treatment was more effective in the patients with a higher neutralizing antibody titer transfusion. More importantly, all the patients in exposure group were survived and discharged, suggesting that the CP treatment was associated with a better outcome and a lower fatality.

In the war of fighting against emerging and pandemic COVID-19, the advantages of the convalescent plasma have been validated by the practice and clinical outcome in China. Therefore, it provided an unprecedented opportunity to perform clinical studies and trials of the efficacy of convalescent plasma transfusion during the pandemic of COVID-19. Meanwhile, the convalescent plasma transfusion is worthy of application and promotion in SARS-CoV-2-infected patients globally due to its safety and efficacy.

## MATERIALS AND METHODS

A retrospective cohort study was conducted from January 22, 2020 to February 28, 2020. 19 patients with COVID-19 in Hunan Province confirmed by real-time viral RNA test and clinical manifestation were hospitalized and received transfusions from the CP donors. To compare the effectiveness of treatment defined by fatality rate, a control group comprised of 43 COVID-19 patients who did not receive CP treatment was selected from Hunan (23 cases) and Hubei (20 cases) provinces using a stratified random sampling method by age, gender and severity of the disease. Baseline characteristics (age, gender, severity, admission date, time from onset to hospitalization, length of days among survivors and time from hospitalization to death) of patients between the exposure group and control group were shown in Supplementary Tables 2, 3. The study was approved by the ethics committees of Xiangya Hospital, Central South University (approval #202002024) and other local hospitals. Written informed consent was obtained from each patient.

## Supplementary Material

Supplementary Tables
