# Supplementary material for: Convalescent plasma to treat COVID-19: clinical experience and efficacy
Source: Aging (Albany NY). 2021 Mar 18;13(6):7758–66. doi: 10.18632/aging.202795 (PMC8034927; doi:10.18632/aging.202795)
Supplement: Supplementary Tables [file aging-13-202795-s001.pdf]

SUPPLEMENTARY TABLES

Supplementary Table 1. Detection of novel coronavirus antibody: Colloidal gold label technology and ELISA.

Sample No.	Colloidal gold label technology		ELISA	
	IgG	IgM	IgG	IgM
1	+	-	640	1280
2	+	-	80	640
3	+	-	210	1280
4	±	-	80	320
5	+	-	160	2560
6	+	-	160	2560
7	±	-	80	-
8	+	-	80	2560
9	-	-	40	320
10	+	-	320	-
11	+	-	80	-
12	±	-	40	-
13	+	-	160	650
14	+	-	160	2560
15	+	-	160	1280
16	±	-	40	-
17	+	±	640	2560
Positive rate	94.1%	5.9%	100%	70.6%

Coincidence Rate: IgG 94.12%.
IgM 29.41%.

Dilution Ratio: Colloidal gold 1:8.
ELISA 1:10.

Supplementary Table 2. Characteristics of the patients of exposure group and control group.

Characteristics	Exposure group	Control group		*P*
	Hunan (N=19)	Hunan (N=23)	Hubei (N=20)	
Age (age), mean±SD	66.3±15.3	57.3±15.0	69.1±14.3	0.030
Sex, n (%)				
Male	11 (57.9)	13 (56.5)	12 (60.0)	0.964
Female	8 (42.1)	10 (35.1)	8 (40.0)	
Severity, n (%)				
Mild-to-moderate	0 (0.0)	0 (0.0)	0 (0.0)	0.053
Severe	6 (31.6)	1 (4.3)	6 (30.0)	
Critical	13 (68.4)	22 (95.7)	14 (70.0)	
Admission date				
First case	22 Jan 2020	20 Jan 2020	3 Feb 2020	
Last case	28 Feb 2020	6 Feb 2020	24 Feb 2020	
Time from onset to hospitalization (days), median (IQR)	4.5 (3.0–7.7)	5.0 (3.0–8.0)	13.0 (8.5–15.0)	<0.001
Length of stay (days) among survivors, median (IQR)	32.5 (24.5–37.7)	20.0 (17.0–21.0)	29.0 (26.0–31.3)	<0.001
Time from hospitalization to death (days), median (IQR)	N/A	10.0 (3.3–22.7)	22.0 (19.0–22.0)	0.157

Supplementary Table 3. The demographic characteristics of the donors.

Donors no.	Blood center	Gender	Age	Donated plasma volume, ml	Blood type	IgM/IgG	Neutralizing antibody titer
D1	Department of Blood Transfusion Laboratory of Changsha Blood Center	Male	40	400	A	Negative/ Positive	80
D3	Department of Blood Transfusion Laboratory of Changsha Blood Center	Male	41	400	A	Positive/ Positive	320
D4	Department of Blood Transfusion Laboratory of Changsha Blood Center	Male	30	400	B	Negative/ Positive	1280
D5	Department of Blood Transfusion Laboratory of Changsha Blood Center	Male	29	400	A	Positive/ Positive	640
D6	Department of Blood Transfusion Laboratory of Changsha Blood Center	Male	38	400	A	Positive/ Positive	1280
D7	Department of Blood Transfusion Laboratory of Zhuzhou Blood Center	Male	47	400	A	Positive/ Positive	320
D8	Department of Blood Transfusion Laboratory of Zhuzhou Blood Center	Male	30	400	A	Positive/ Positive	640
D9	Department of Blood Transfusion Laboratory of Zhuzhou Blood Center	Female	26	400	O	Positive/ Positive	160
D10	Department of Blood Transfusion Laboratory of Zhuzhou Blood Center	Female	41	400	A	Did not detected	Did not detected
D11	Department of Blood Transfusion Laboratory of	Female	44	400	A	Negative/ Positive	640

	Zhuzhou Blood Center						
D12	Department of Blood Transfusion Laboratory of Zhuzhou Blood Center	Female	23	400	AB	Negative/ Positive	640
D13	Department of Blood Transfusion Laboratory of Zhuzhou Blood Center	Male	31	400	AB	Positive/ Positive	320
D15	Department of Blood Transfusion Laboratory of Yueyang Blood Center	Female	49	300	B	Positive/ Positive	640
D16	Department of Blood Transfusion Laboratory of Yueyang Blood Center	Male	43	400	O	Positive/ Positive	640
D17	Department of Blood Transfusion Laboratory of Yueyang Blood Center	Male	22	400	O	Positive/ Positive	640
D20	Department of Blood Transfusion Laboratory of Loudi Blood Center	Female	36	400	O	Positive/ Positive	1280
D21	Department of Blood Transfusion Laboratory of Loudi Blood Center	Female	38	400	A	Weekly Positive/ Positive	320
D23	Department of Blood Transfusion Laboratory of Loudi Blood Center	Male	41	400	O	Weekly Positive/ Weekly Positive	80
D24	Department of Blood Transfusion Laboratory of Loudi Blood Center	Male	42	400	A	Positive/ Positive	320
D25	Department of Blood Transfusion Laboratory of Loudi Blood Center	Male	40	400	B	Weekly Positive/ Positive	320
D27	Department of Blood Transfusion Laboratory of Xiangtan Blood Center	Male	47	400	O	Positive/ Positive	640
D30	Department of Blood Transfusion Laboratory of Shaoyang Blood Center	Male	24	300	A	Did not detected	Did not detected
D33	Department of Blood Transfusion Laboratory of Shaoyang Blood Center	Female	22	200	A	Negative/ Positive	320
